# Supplementary material for: The Antifungal Properties of Tamarix aphylla Extract against Some Plant Pathogenic Fungi
Source: Microorganisms. 2023 Jan 4;11(1):127. doi: 10.3390/microorganisms11010127 (PMC9861458; doi:10.3390/microorganisms11010127)
Supplement: Supplementary file 1 [file microorganisms-11-00127-s001.zip › microorganisms-2041971-Supplementary materials.pdf]

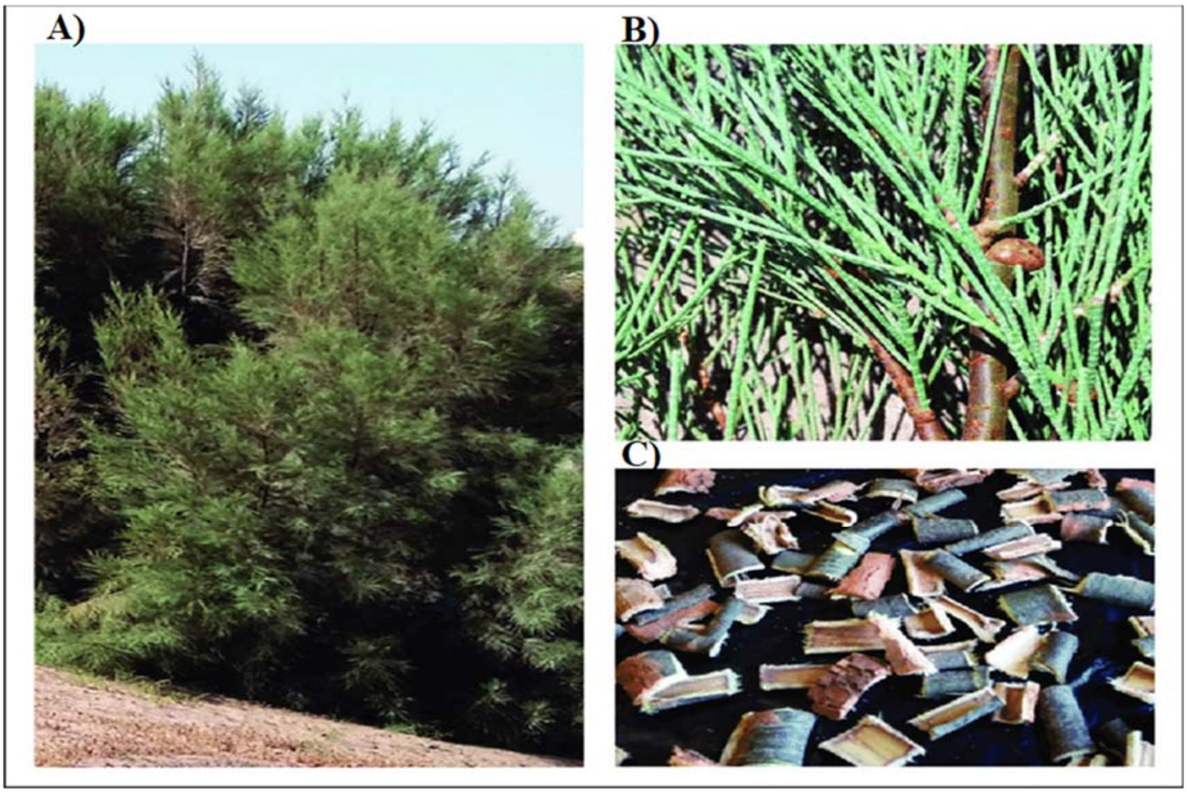

Figure S1: photographs of *T. aphylla* (L.) Karst. (Saudi species): (A) habit: whole tree, (B) branches with leaves, and (C) dried bark.
